# LMT AMI Caused by a Valve-Like Ridge in a 9-Year-Old Boy Successfully Treated With PCI

**DOI:** 10.1016/j.jaccas.2024.102404

**Published:** 2024-06-14

**Authors:** Kotaro Takahashi, Tomohisa Tada, Mistuhiko Yahata, Kiyotaka Shimamura, Ryuusuke Nishikawa, Philip Hawke, Nobuhiro Matsuyama, Junji Sakata, Yasuyo Takeuchi, Makoto Motooka, Mizuhiko Ishigaki, Sung-Hae Kim, Hiroki Sakamoto

**Affiliations:** a Department of Cardiology, Shizuoka General Hospital, Shizuoka, Japan; b School of Pharmaceutical Sciences, University of Shizuoka, Shizuoka, Japan; c Department of Cardiology, Shizuoka Children's Hospital, Shizuoka, Japan

**Keywords:** Acute Coronary Syndrome, coronary angiography, intravascular ultrasound

## Abstract

A 9-year-old boy was suspected of having acute myocardial infarction and emergency coronary angiogram was performed. No signs of flow limitation in either coronary artery was detected. We performed intravascular ultrasonography from the ascending aorta, which showed a ridge on the left main trunk acting like a valve, resulting in significant stenosis. Percutaneous coronary intervention with stent deployment was performed with good result.

## Graphical abstract

**Figure:**
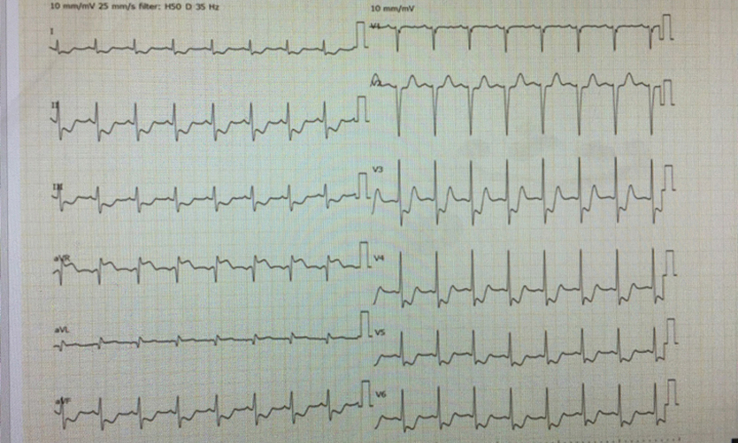

## History of Presentation

**Figure 1:**
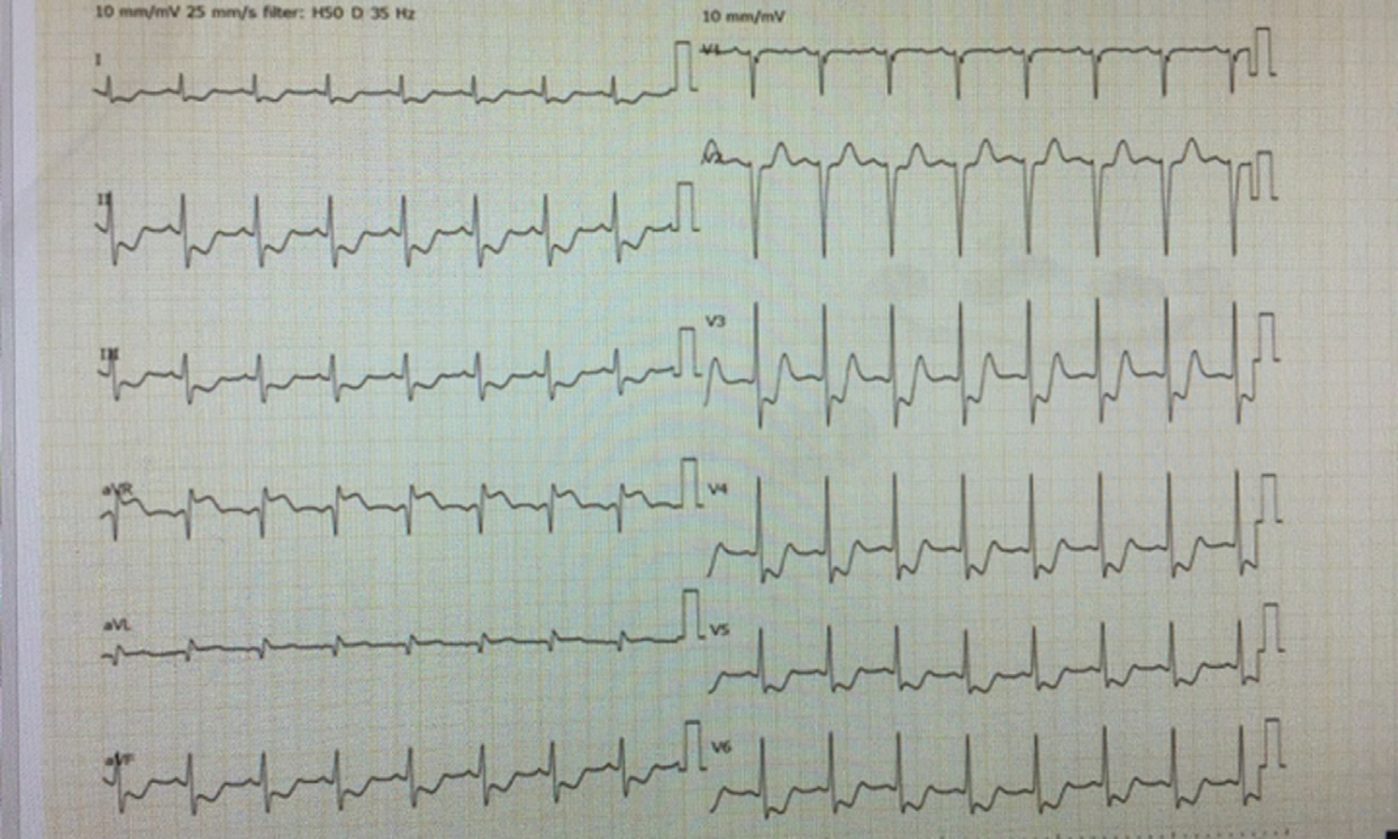
Initial Electrocardiogram Heart rate128 beats/min, ST-segment elevation in augmented vector of the right arm, and ST-segment depression in precordial leads.

A 9-year-old boy presented to our hospital with ongoing chest pain. Electrocardiography revealed ST-segment elevation in augmented vector of the right arm and ST-segment depression in precordial leads suggestive of left main trunk (LMT) acute myocardial infarction (Figure 1), and emergency coronary angiography (CAG) was performed.

**Learning Objectives**

• To understand the pathophysiology of acute coronary syndrome caused by valve-like ridge ostium of LMT using intravascular imaging.
• To understand that there is a possibility of overlooking stenosis in coronary ostium when engaging a catheter into the coronary, whereas nonselective left coronary sinus angiography may have chance of clarifying the underlying disease.

## Medical History

The patient had a history of bronchial asthma, and no asthma attacks were detected for years, and had not taken any medication.

## Differential Diagnosis

A possible cause of chest pain and electrocardiography findings are takotsubo cardiomyopathy and acute myocarditis with pericarditis, but, because Acute Coronary Syndrome is an emergent condition, CAG had to be performed immediately.

## Investigations

**Figure 2:**
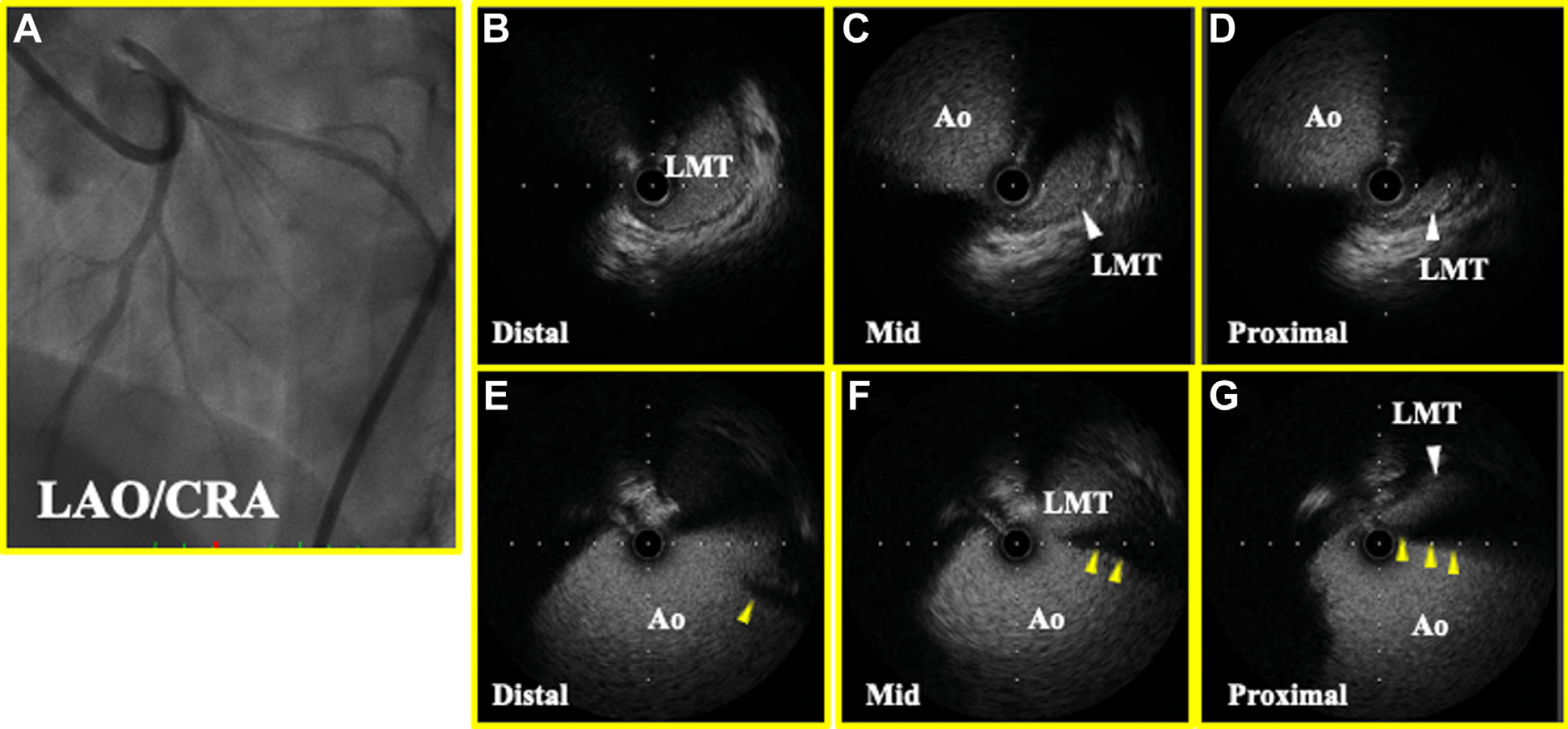
Enhanced Angiogram and IVUS (A) Enhanced angiogram from LCC of LAD, showing slit-like appearance in ostium of LMT (yellow arrowhead). (B to D) IVUS from LMT showing black shadowing, indicating valve-like ridge (yellow arrowhead). (E to G) IVUS from ascending aorta. Ao = aorta; CRA = cranial position; IVUS = intravascular ultrasound; LAD = left anterior descending artery; LAO = left anterior oblique position; LCC = left coronary cusp; LMT = left main trunk.

CAG revealed several collateral arteries extended from the right coronary artery to the left anterior descending artery, but there were no signs of flow limitation in either coronary artery (Video 1). The patient was transferred to cardiac intensive care. The next day, when chest pain recurred with cold sweats and increases in levels of creatinine kinase (4559) and creatinine kinase-myocardial band (382), emergency CAG was performed again, achieving the same results as the initial procedure. Nonselective left coronary sinus angiography revealed a slit at the ostium of the LMT, suggesting severe stenosis (Figure 2A), and percutaneous coronary intervention (PCI) was performed.

## Management

**Figure 3:**
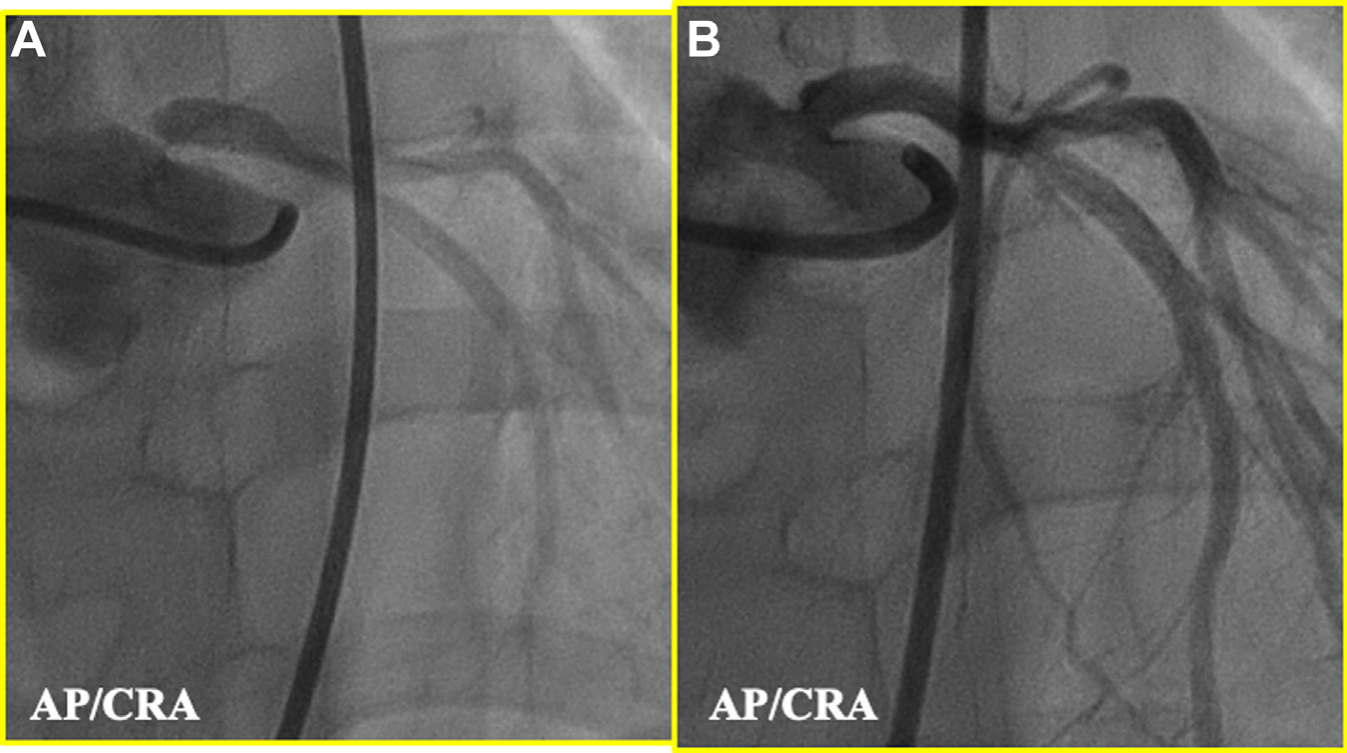
Enhanced Angiogram (A) Enhanced angiogram from LCC after first stent. Slit-like appearance still persists (yellow arrowhead). (B) Enhanced angiogram from LCC after second stent shows that the stent has covered the stenosis and slit-like appearance cannot further be detected. AP = anterior posterior position; other abbreviations as in Figure 2.

Intravascular ultrasonography from the ascending aorta showed a ridge on the LMT acting like a valve, resulting in significant stenosis (Figures 2B to 2G). Attempted implantation of a bare metal stent (3.5/8 mm) did not fully cover the ostium (Figure 3A), but a second bare metal stent (3.5/8 mm) successfully covered it (Figure 3B).

## Discussion

**Figure 4:**
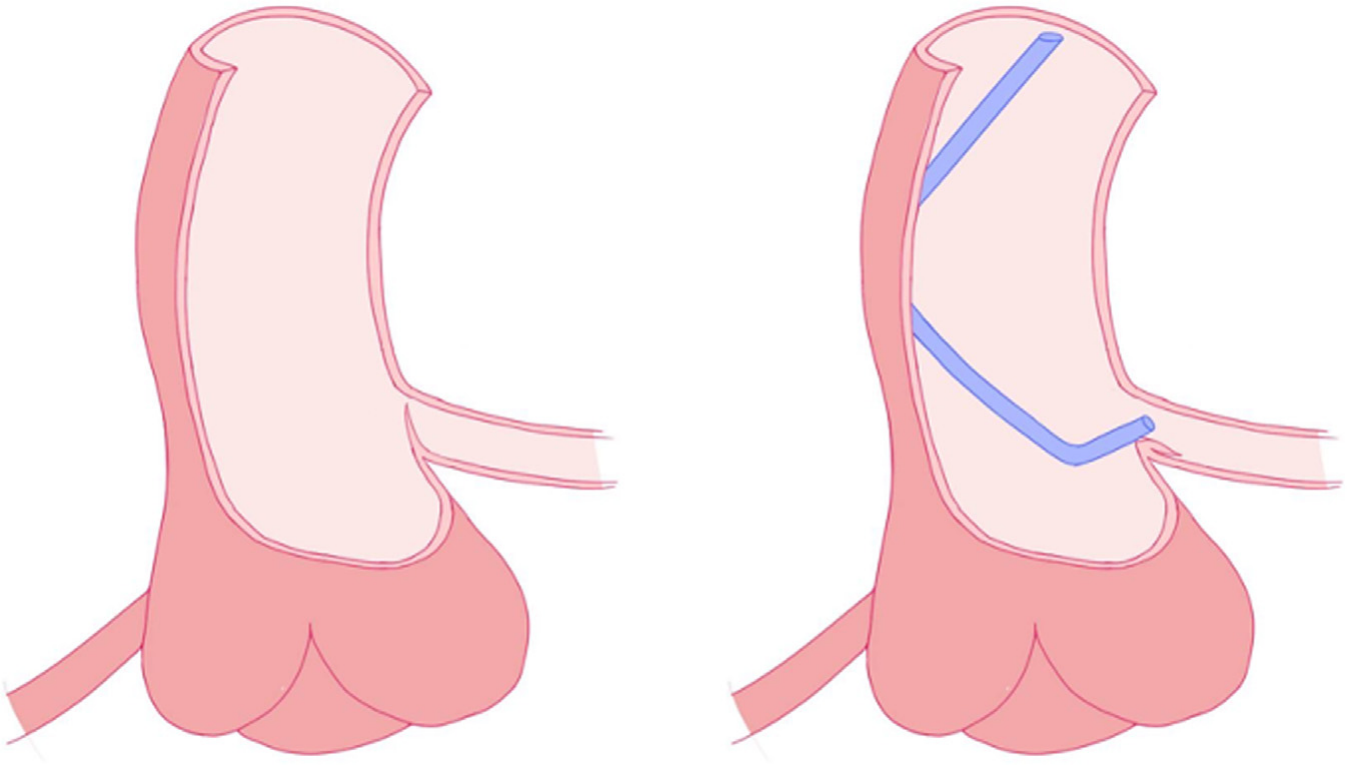
Schema Schema of ostium with valve-like ridge (left) and ridge pushed against coronary wall by catheter (right).

There are several reports of a valve-like ridge on the ostium of the LMT resulting in ischemia and sudden death, but this appears to be the first report of successful treatment with PCI. The condition has poor prognosis, perhaps due to difficulty in diagnosis because a standard angiogram may not reveal the ridge when the catheter pushes it against the coronary wall (Figure 4). In this case, the flap created by the ridge was identified with a nonselective left coronary sinus angiography and intravascular ultrasonography from the aorta. We suppose that the non-selective left coronary sinus angiography is useful method, when usual coronary angiography by engaging catheter into coronary artery could not identify the culprit lesion, in case of AMI caused by valve-like ridge ostium. The stent covering the ridge performed ideally, both short- and long-term.

## Follow-up

The patient was prescribed aspirin 100 mg and prasugrel 3.75 mg for the 3 months after the PCI, followed by aspirin 100 mg alone after that. He is doing well 7 years after the primary event.

## Conclusions

Acute myocardial infarction caused by valve-like ridge ostium is known to have poor prognosis, possibly due to difficulty in diagnosing the disease, whereas use of an intravascular imaging device and nonselective coronary sinus angiography made it possible to diagnose this condition while the patient is alive. PCI may be a good choice for these patients with good prognosis.

## Funding Support and Author Disclosures

The authors have reported that they have no relationships relevant to the contents of this paper to disclose.

## Appendix

Video 1 Initial CAG of right coronary artery.

Video 2 Initial CAG of left coronary artery.

